# PIWIs Go Viral: Arbovirus-Derived piRNAs in Vector Mosquitoes

**DOI:** 10.1371/journal.ppat.1006017

**Published:** 2016-12-29

**Authors:** Pascal Miesen, Joep Joosten, Ronald P. van Rij

**Affiliations:** Department of Medical Microbiology, Radboud Institute for Molecular Life Sciences, Radboud University Medical Center, Nijmegen, The Netherlands; University of Alberta, CANADA

## Abstract

Vector mosquitoes are responsible for transmission of the majority of arthropod-borne (arbo-) viruses. Virus replication in these vectors needs to be sufficiently high to permit efficient virus transfer to vertebrate hosts. The mosquito immune response therefore is a key determinant for arbovirus transmission. Mosquito antiviral immunity is primarily mediated by the small interfering RNA pathway. Besides this well-established antiviral machinery, the PIWI-interacting RNA (piRNA) pathway processes viral RNA into piRNAs. In recent years, significant progress has been made in characterizing the biogenesis and function of these viral piRNAs. In this review, we discuss these developments, identify knowledge gaps, and suggest directions for future research.

## Small RNAs in Arboviral Infections

Mosquitoes and other hematophagous arthropods transmit important human and animal viruses, some of which are responsible for debilitating diseases such as dengue, chikungunya, and Zika [1]. Collectively, this nontaxonomical group of viruses is termed arthropod-borne viruses (arboviruses). Most arboviruses are RNA viruses with either double-stranded RNA (dsRNA) genomes or single-stranded RNA (ssRNA) genomes of positive (+) or negative (-) polarity. The majority can be assigned to the families *Bunyaviridae* (-ssRNA), *Flaviviridae* (+ssRNA), *Reoviridae* (dsRNA), *Rhabdoviridae* (-ssRNA), and *Togaviridae* (+ssRNA) [2]. Because of an increased incidence and expansion of the geographical range of anthropophilic vector mosquitoes, the global threat of arboviruses is increasing [1,3]. Interestingly, while having the potential to cause severe disease in vertebrate hosts, arboviruses replicate to high levels in their mosquito vectors without causing apparent pathology [4,5]. This suggests that vector mosquitoes possess efficient mechanisms to resist or tolerate virus infection, despite lacking the adaptive immune system and interferon-mediated antiviral responses of vertebrates [6].

Whereas the evolutionary conserved Toll, Imd, and Jak-Stat signaling pathways are implied in antiviral defense [7], the cornerstone of antiviral immunity in insects is believed to be the small interfering RNA (siRNA) pathway [8,9]. This pathway is initiated by cleavage of viral dsRNA into 21-nucleotides (nt)-long siRNAs by the RNase-III endonuclease Dicer-2 [10,11]. These siRNAs associate with Argonaute 2 (Ago2) in an RNA-induced silencing complex (RISC) and serve as a guide for Ago2-mediated cleavage of viral target sequences [10,12]. Accordingly, experimental inactivation of siRNA pathway components in mosquitoes results in increased arbovirus replication [13–18]. The fact that several insect viruses have evolved suppressors of the siRNA pathway underlines its importance in antiviral immunity [8,19]. Likewise, arboviral gene products have been proposed to act as antagonists of the siRNA pathway in mosquitoes [20–22].

MicroRNAs comprise an independent class of small RNAs that may be involved in the cellular response to arboviral infections by regulation of host immune genes [23]. They are produced from genome-encoded stem-loop RNA structures in a Dicer-1- and Ago1-dependent manner, akin to siRNA biogenesis [24]. The role of siRNAs and microRNAs in mosquito–arbovirus interactions is beyond the scope of this review and is discussed extensively elsewhere [8,9,23,25].

In this review, we will focus on the most enigmatic class of small silencing RNAs in the context of arbovirus–vector interactions: PIWI-interacting (pi)RNAs. piRNAs associate with the PIWI clade of the Argonaute protein superfamily, display a broad size range (24–30 nt), and are produced independently of Dicer [26]. The canonical function of the piRNA pathway is protection of genome integrity in animal germ cells by silencing transposons, selfish genetic elements with the ability to randomly integrate into the host genome [27]. Recently, however, several groups, including ours, have reported de novo production of piRNAs derived from viral sequences in the vector mosquitoes *Aedes aegypti* and *Ae*. *albopictus* and in cell lines derived from these animals [28–39]. Biogenesis of viral piRNAs (vpiRNAs) occurs independent of siRNA production, which raises the exciting possibility that vpiRNAs may constitute an additional line of defense against arboviruses in vector mosquitoes.

**Fig 1:**
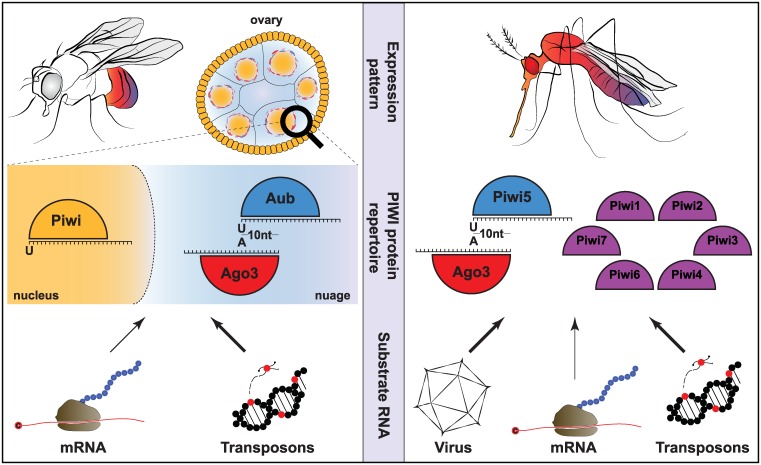
Divergence of piRNA pathways in *Drosophila melanogaster* and *Aedes aegypti*. In *Drosophila* (left panel), PIWI proteins are almost exclusively expressed in gonadal tissues. Nuclear Piwi is expressed in both germ cells and ovarian somatic cells, whereas Aub and Ago3 expression is limited to germ cells specifically. In the nuage surrounding the nucleus of these cells, Aub and Ago3 form the ping-pong amplification complex, which is responsible for secondary piRNA production with the characteristic 1U/10A nucleotide bias (Box 1). *Drosophila* piRNAs are mainly derived from transposon sequences and to a lesser extent from mRNA. In *Ae*. *aegypti* (right panel), the PIWI protein family is expanded to eight members (Piwi 1–7 and Ago3), some of which are expressed in somatic tissues. Of these PIWI proteins, Piwi5 and Ago3 interact to produce piRNAs with the 1U/10A nucleotide bias indicative of secondary piRNA production through ping-pong amplification. In *Aedes*, piRNAs are produced from viral RNA, in addition to transposon sequences and mRNA.

Our understanding of the piRNA pathway in insects is incomplete and largely biased towards studies in the genetic model insect *Drosophila melanogaster* (Box 1). Yet, piRNA pathways in vector mosquitoes differ considerably from *Drosophila* and other model organisms. This becomes apparent in many aspects: (i) The composition of piRNA pathway components differs between *Drosophila* and mosquitoes (Fig 1). Notably, the PIWI gene family, which lies at the heart of the piRNA pathway, has undergone expansion in both *Aedes* and *Culex* mosquitoes [40,41]. In addition, the recent annotations of mosquito genomes do not contain orthologs for all the established factors involved in *Drosophila* piRNA biogenesis and function [42]. (ii) Mosquito PIWI proteins have an extended expression pattern (Fig 1). For instance, some of the members of the expanded *Aedes* PIWI family are expressed in somatic tissue [43], whereas expression of PIWI proteins in *Drosophila* is largely restricted to gonadal tissues [44–47]. (iii) The piRNA pathway in *Aedes* processes a broader repertoire of substrates (Fig 1). Despite the large transposon content of the *Ae*. *aegypti* genome [48], relatively few piRNAs are derived from these mobile elements [49]. Instead, a considerable proportion of piRNAs are derived from nonrepetitive genomic areas, including the open reading frames of protein-coding genes [49]. Yet, the most prominent gain of function is the production of piRNAs from viral RNA during the course of an acute infection.

**Box 1. piRNA Biogenesis in *Drosophila***

In the *Drosophila* germline, the mobilization of transposable elements is efficiently suppressed by transcriptional and posttranscriptional gene silencing by the piRNA pathway. piRNA biogenesis involves the primary processing pathway and ping-pong amplification that is capable of triggering phased piRNA production. Below, we provide a brief description of the *Drosophila* piRNA pathway; for a comprehensive review, we refer to [26,50].

During primary processing, single-stranded piRNA precursors are generated from genomically encoded piRNA clusters that are rich in transposon remnants [44]. The endonuclease Zucchini (Zuc) cleaves these precursors directly upstream of uridine residues, thus producing piRNA intermediates with a bias for a uridine at the first nucleotide position (1U) [51–53]. In an electron-dense perinuclear structure termed nuage, these piRNA intermediates are loaded onto the PIWI proteins Piwi and Aubergine (Aub). Once bound, piRNA intermediates are trimmed and 2′-*O* methylated at their 3′ end, forming mature piRNAs [54–57]. Mature piRNA-loaded Piwi translocates to the nucleus and associates with Asterix and Panoramix/Silencio for transcriptional silencing of transposons through deposition of repressive chromatin marks [58–63].

piRNA-loaded Aub remains in the nuage where it initiates the secondary ping-pong amplification cycle by recognition and cleavage of cognate transposon mRNA [44,45,64]. The resulting cleavage product forms the precursor of a secondary sense piRNA that associates with Ago3. piRNA-loaded Ago3 can target and cleave antisense piRNA precursors generating the 5′ end of new sense piRNAs that can be loaded onto Aub, completing the ping-pong amplification cycle [44,45].

Recent work has demonstrated a preference for uridine at the 5′ position in the binding pocket of the MID (middle) domain of PIWI proteins [65,66]. In combination with the predisposition of Zuc to cleave directly 5′ of uridine residues, this causes Aub to associate predominantly with 1U antisense piRNAs. A subset of PIWI proteins, including Aub and silkworm Siwi, have an additional preference for target RNAs carrying an adenosine directly opposite of the first position of the piRNA [66,67]. As PIWI-mediated cleavage occurs specifically between nucleotide 10 and 11, Ago3-associated sense piRNAs are enriched for adenosine residues at their tenth position (10A). The resulting 1U/10A signature is a characteristic hallmark of secondary ping-pong amplification of piRNAs. Secondary amplification endows the piRNA pathway with specificity, as from a diverse pool of primary piRNAs, only those recognizing active transposons are amplified.

Recent studies have proposed that secondary piRNAs initiate Zuc-dependent production of phased piRNAs [52,53]. Cleavage by Zuc determines the 3′ termini of Aub-associated piRNAs, while the downstream fragment is processed further into Piwi-associated piRNAs by successive Zuc-mediated cleavage events [68]. These piRNAs show ~27 nt phasing and a strong 1U bias because of the preference of Zuc to cleave upstream of uridine residues. Phased piRNA production increases the diversity of the piRNA pool and allows adaptation of the piRNA pathway to changes in transposon sequence.

## vpiRNAs in *Aedes* Mosquitoes

Initial evidence for vpiRNA production came from the analysis of small RNA deep-sequencing data of the *Drosophila* ovarian somatic sheet (OSS) cells persistently infected with several RNA viruses [69]. OSS cells exclusively express Piwi but lack the PIWI proteins that act in the ping-pong amplification machinery. Since Piwi preferentially associates with piRNAs containing a uridine at the first nucleotide position, both sense and antisense vpiRNAs produced in these cells bear a 1U bias (Table 1). However, to date, vpiRNAs have never been found in adult flies. Even infection with Sigma virus, which naturally infects *Drosophila* germ cells, does not give rise to vpiRNA production [70], despite ample expression of PIWI proteins in these cells. In sharp contrast, vpiRNAs are readily detected both in *Aedes* cell lines and in somatic tissues of adult *Aedes* mosquitoes upon infection with several arboviruses, including members of the *Togaviridae* [28–33], *Flaviviridae* [34–36], *Bunyaviridae* [28,29,37–39], and *Reoviridae* [37] (Table 1). Besides a typical size distribution of small RNAs around 24–30 nt, piRNAs from several viruses display the characteristic nucleotide bias indicative of ping-pong amplification (Box 1). Across all virus families, the secondary 10A-biased piRNAs are enriched for the strand with coding capacity, yet the mechanisms responsible for this sorting remain elusive. In addition, vpiRNAs from dengue virus (*Flavivirus* genus, *Flaviviridae* family) and Sindbis virus (*Alphavirus* genus, *Togaviridae* family) have been verified to be 2′-*O* methylated at the 3′ terminal nucleotide (Table 1), a modification that is present on all PIWI-loaded mature piRNAs (Box 1). PIWI-dependence of vpiRNAs has been established for dengue, Sindbis, and Semliki Forest virus (*Alphavirus* genus, *Togaviridae* family) [31,32,36] and direct association with PIWI proteins has been demonstrated for Sindbis virus–derived piRNAs [32].

**Table 1:** vpiRNA production in infections with arboviruses and insect-specific viruses.

Virus family	Name	Genus	Genome	Host and cells*	Nucleotide and (strand) biases**	3′ end modification	PIWI protein-dependent	Reference
*Togaviridae*	**Sindbis virus**	*Alphavirus*	+ssRNA	Aag2, U4.4, C6/36	1U (-), 10A (+)	yes	Piwi5/Ago3 in Aag2 cells***	[28,29,32]
	**chikungunya virus**	*Alphavirus*	+ssRNA	*Ae*. *aegypti; Ae*. *albopictus (soma);* U4.4, C6/36, C7-10	1U (-), 10A (+)	n.a.	n.a.	[30,33]
	**Semliki Forest virus**	*Alphavirus*	+ssRNA	Aag2, U4.4	1U (-), 10A (+)	n.a.	Loss of vpiRNAs upon combined knockdown of Piwi1-7 and Ago3 in Aag2 cells	[31]
*Flaviviridae*	**dengue virus, serotype 2**	*Flavivirus*	+ssRNA	*Ae*. *aegypti;* Aag2, C6/36	10A (+)	yes	Piwi5, Ago3, and to a lesser extent Piwi6 in Aag2 cells	[34–36]
	**cell fusing agent virus**	*Flavivirus*	+ssRNA	Aag2, C6/36	10A (+)	n.a	n.a.	[34]
*Bunyaviridae*	**La Crosse virus**	*Orthobunyavirus*	-ssRNA, 3 segments	C6/36	1U (-), 10A (+)	n.a.	n.a.	[28,29]
	**Schmallenberg virus**	*Orthobunyavirus*	-ssRNA, 3 segments	KC, Aag2	1U (-), 10A (+)	n.a.	n.a.	[37]
	**Rift Valley fever virus**	*Phlebovirus*	-ssRNA, 3 segments	Aag2, U4.4, C6/36	1U (-), 10A (+)	n.a.	n.a.	[38]
	**Phasi Charoen-like virus**	unclassified	-ssRNA, 3 segments	*Ae*. *aegypti*	1U (-), 10A (+)	n.a.	n.a.	[39]
*Reoviridae*	**bluetongue virus**	*Orbivirus*	dsRNA10 segments	KC, Aag2	n.a.	n.a.	n.a.	[37]
*Dicistroviridae*	**Drosophila C virus**	*Cripavirus*	+ssRNA	OSS	1U	n.a.	n.a.	[69]
*Nodaviridae*	**American nodavirus**	*Alphanodavirus*	+ssRNA, 2 segments	OSS	1U	n.a	n.a.	[69]

n.a., not analyzed.

*Aag2 cells are derived from *Ae*. *aegypti* mosquitoes; U4.4, C6/36, and C7-10 cells are derived from *Ae*. *albopictus* mosquitoes; KC cells are derived from *Culicoides sonorensis;* OSS cells are derived from the ovarian somatic sheet of *Drosophila melanogaster*.

** The strand orientation is defined in relation to translation; (+) refers to the sense strand with coding potential, (-) refers to the antisense strand. For ssRNA viruses, this reflects the antigenome and genome, respectively.

*** (+) strand piRNAs associate with Ago3 and (-) strand piRNAs associate with Piwi5.

## Determinants of vpiRNA Biogenesis

The substrate for the antiviral siRNA pathway, double-stranded RNA, is not abundant in the cytoplasm of healthy, uninfected cells and therefore serves as a danger signal indicating ongoing virus infection [71]. In contrast, the substrate for vpiRNA biogenesis is a single-stranded viral RNA. It is unknown how PIWI proteins distinguish viral from host RNA and how they determine which of these transcripts are used for piRNA biogenesis. Like cellular mRNAs, single-stranded (+) RNAs of major arbovirus families carry a 5′ cap, produced by a virus-encoded capping machinery (flaviviruses and alphaviruses) or through a mechanism termed cap-snatching (bunyaviruses) [72]. In contrast to the eukaryotic and flavivirus capping machineries, that of alphaviruses does not deposit 2′-*O* methylation marks at the first two nucleotides downstream of the cap structure [72,73]. Additionally, genomic RNAs of flaviviruses lack the poly-A tail normally present on cellular mRNAs [74]. In analogy to innate immune sensors of vertebrates, it is conceivable that the mosquito PIWI proteins specifically recognize such nonself RNA features or that they are recruited to these features by adaptor proteins.

**Fig 2:**
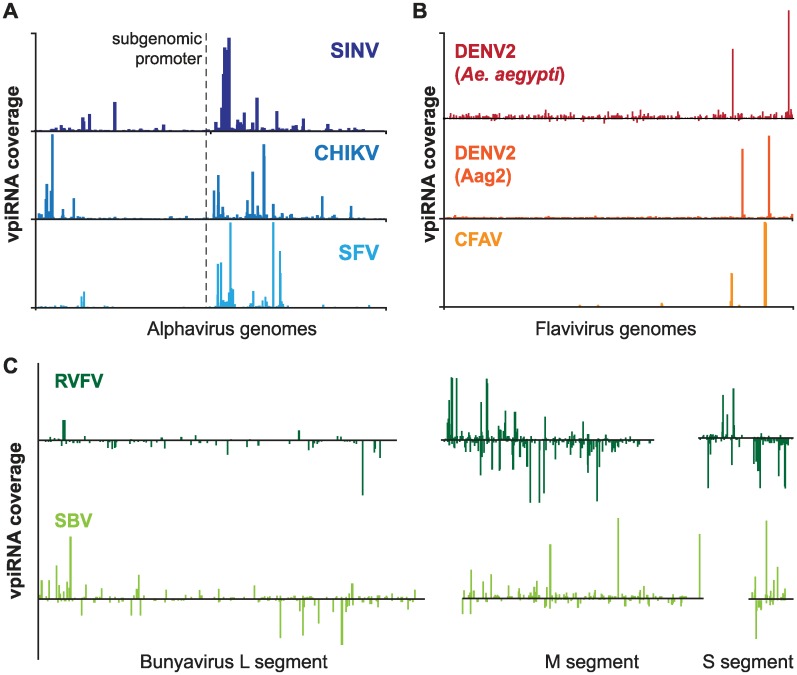
Viral piRNA profiles. piRNA distributions across the genomes of selected (A) alphaviruses, (B) flaviviruses, and (C) bunyaviruses. The plots depict published genome profiles of Sindbis virus (SINV) [29], chikungunya virus (CHIKV) [30], Semliki Forest virus (SFV) [31], dengue virus serotype 2 (DENV2) [35,36], cell fusing agent virus (CFAV) [34], Rift Valley fever virus (RVFV) [38], and Schmallenberg virus (SBV) [37]. For alphaviruses, the position of the subgenomic promoter is depicted. The piRNA coverage on the sense or antisense strand is shown as peaks above or below the *x*-axis, respectively. Please note that the plots are representations of piRNA profiles from multiple studies that used different ways of normalizing and presenting read counts. Therefore, the heights of the bars are arbitrary and do not allow a quantitative comparison between the different viruses.

A clue that may help in understanding the mechanisms of target selection lies in the genomic distribution of vpiRNAs. While approximately equal levels of viral siRNAs (vsiRNAs) are produced along arbovirus genomes, vpiRNA production is mostly confined to specific hotspot regions. In alphaviruses such as Sindbis, chikungunya, and Semliki Forest virus, vpiRNAs are predominantly produced from a subgenomic RNA that is transcribed from an internal promoter sequence (Fig 2A). This may be due to higher expression of subgenomic relative to genomic RNA. For example, for Sindbis virus it has been shown that the subgenomic promoter yields an excess of subgenomic RNA compared to full length genomic RNA [75,76]. Furthermore, subgenomic ssRNA may be more accessible for the piRNA machinery because it is required for translation of the structural proteins at later stages of the infection. However, these hypotheses do not explain why alphavirus-derived piRNAs are not uniformly distributed over the length of the subgenomic RNA but rather display very discrete hotspots in the 5′ region of the capsid gene (Fig 2A). One mechanism that could underlie this pattern is processing of abortive viral RNA transcripts by the piRNA machinery. Incomplete viral transcripts are not protected by RNA replication or translation machineries and may therefore represent easily accessible substrates for vpiRNA production. Alternatively, RNA sequences or structural elements may recruit piRNA biogenesis factors to specific regions of the viral genomes. Recently, Homolka et al. described such a piRNA-trigger sequence (PTS) in the *Drosophila flamenco* locus, which evokes piRNA biogenesis independent of its genomic context. However, whether this PTS is a structural motif or harbors a small, as-yet unrecognized sequence motif remains to be unraveled [77]. Similarly, Ishizu et al. identified a *cis*-acting, 100-nt fragment in the 3′UTR of the piRNA-producing gene *traffic jam* that triggers piRNA production when expressed from unintegrated plasmid DNA. These plasmid-derived piRNAs were efficient in transcriptional silencing of endogenous genes [78]. In light of these data, it would be interesting to test whether vpiRNA hotspot sequences promote piRNA production when placed outside their viral context.

piRNA hotspots in flavivirus genomes, including dengue and cell fusing agent virus, differ considerably from those in alphaviruses. Flavivirus piRNAs mostly derive from few very discrete hotspots, sometimes representing single sequences (Fig 2B). The nature of these piRNA spikes remains obscure, but this difference strongly suggests that the mechanisms underlying alphavirus and flavivirus piRNA biogenesis are fundamentally different.

Common to alphavirus and flavivirus piRNAs is their extreme strand bias towards sequences from the viral sense strands. In sharp contrast, bunyavirus piRNAs are produced from both antigenomic and genomic strands at a more equal ratio (Fig 2C). It is currently unclear whether this reflects differences in the replication strategies of alphaviruses and flaviviruses (both +ssRNA viruses) compared to bunyaviruses (-ssRNA virus) or if this is due to variations in the piRNA machinery acting on RNAs of distinct viruses. These observations clearly underscore the need for a comprehensive analysis of *cis*- and *trans*-acting factors required for the piRNA biogenesis from arboviruses of all families.

## Biogenesis of vpiRNAs

**Fig 3:**
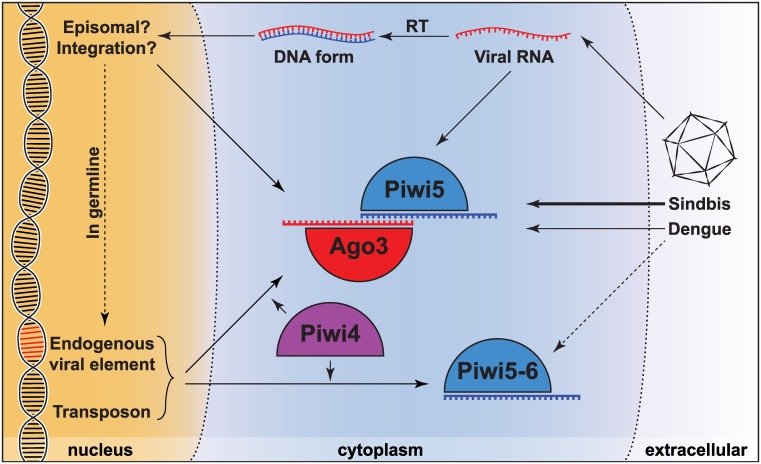
Model for piRNA biogenesis in *Aedes aegypti*. RNA molecules from varying sources are processed differently by the piRNA machinery in *Ae*. *aegypti*. Upon acute infection, Sindbis virus RNA is processed into ping-pong–dependent piRNAs involving PIWI proteins Piwi5 and Ago3. In contrast, dengue virus RNA can also be processed into piRNAs by Piwi6. Transposon-derived piRNAs associate primarily with Piwi5 and Piwi6; however, some transposon RNAs feed into the ping-pong loop and give rise to Ago3-bound secondary piRNAs. Additionally, the production of transposon piRNAs is dependent on Piwi4 in an indirect manner, as transposon-derived piRNAs are not loaded in Piwi4, but knockdown of *Piwi4* does reduce their numbers. Viral RNA may directly enter the piRNA machinery; additionally, viral RNA is reverse transcribed to produce a DNA form of the virus (vDNA). The vDNA may either remain episomal or integrate into the host genome. Putative vDNA-derived transcripts may serve as additional precursors for vpiRNA production. Moreover, when genome integration occurs in the germline, the vDNA fragment forms a novel endogenous viral element (EVE) that may lead to the production of EVE-derived piRNAs.

Functional diversification of *Aedes* PIWI proteins after gene duplication in combination with somatic expression are likely the main drivers of the expansion of piRNA substrates, including viral RNA. *Ae*. *aegypti Piwi4*, *Piwi5*, *Piwi6*, and *Ago3* are abundantly expressed in somatic tissue of adult mosquitoes [43] and *Ae*. *aegypti* Aag2 cells [29]. In an RNAi screen targeting individual PIWI proteins in Aag2 cells, our group identified *Piwi5* and *Ago3* as the main players for vpiRNA production from Sindbis virus. Piwi5 and Ago3 bind vpiRNAs from opposite strands and with distinct nucleotide biases. Whereas Piwi5 binds 1U-biased antisense piRNAs, Ago3 binds 10A-biased piRNAs derived from the viral sense strand [32]. These observations suggest a model in which ping-pong amplification is initiated by Piwi5-bound primary piRNAs from the Sindbis virus antisense strand. Cleavage of the sense strand by Piwi5 results in the production of secondary sense strand piRNAs that are loaded into Ago3 (Fig 3).

Knockdown of *Piwi5* and *Ago3*—and to a lesser extent, *Piwi6*—results in reduced vpiRNA production from dengue virus serotype 2 in Aag2 cells [36]. The additional requirement of Piwi6 specifically for dengue virus piRNA biogenesis suggests that *Aedes* PIWI proteins have specialized in processing distinct RNA sources. This is further supported by the differential requirement of PIWI proteins for the processing of transposon-derived piRNAs that, in contrast to Sindbis virus–derived piRNAs, directly or indirectly relies on all somatic *Aedes* PIWI proteins [32] (Fig 3). Future research should define to what extent vpiRNA production relies on similar or distinct PIWI family members for viruses within the same virus family and between different virus families. Of special interest are bunyaviruses, for which PIWI dependency thus far has not been studied, despite the fact that these viruses represent the largest arbovirus family [2].

## The piRNA Pathway Acts in Resistance and Tolerance to Virus Infections

Arboviruses establish persistent infections in mosquitoes without causing apparent fitness loss in their vectors, despite high viral load. Such a defense strategy in which high pathogen levels are tolerated and the focus lies on preventing infection-induced damage has been termed tolerance. In contrast, actively restricting virus growth and potentially clearing the infection is a defense strategy called resistance [6]. Although a comprehensive model for vpiRNA function is still lacking, there is good evidence that the piRNA pathway is implicated in both strategies.

For example, it was shown that upon knockdown of *Piwi4* in *Ae*. *aegypti* Aag2 cells, replication of Semliki Forest virus is strongly enhanced [31]. Yet, this resistance seems to be independent of vpiRNA production, as Piwi4 depletion does not cause reduction of vpiRNA levels [31]. In line with this observation, immunoprecipitation of Piwi4 in Aag2 cells infected with a related alphavirus (Sindbis virus) is depleted of vpiRNAs [32]. Therefore, the molecular mechanism by which Piwi4 exerts its antiviral activity remains to be investigated. Knockdown of *Piwi5* and *Ago3* in Aag2 cells results in profound decline in vpiRNA expression from Sindbis virus and dengue virus, but viral replication is not strongly affected [36]. Whether PIWI depletion in adult mosquitoes causes enhanced arbovirus replication remains to be shown.

Interestingly, in mosquito cells infected with Rift Valley fever virus (*Phlebovirus* genus, *Bunyaviridae* family), vpiRNAs are primarily detected late in infection following a first wave of vsiRNAs. The vpiRNAs vastly outnumber vsiRNAs at 72 hours postinfection [38]. These data suggest that vpiRNAs may exert their function primarily late during Rift Valley fever virus infection or during the establishment of a persistent infection. Similarly, Goic et al. show that ping-pong–amplified piRNAs are present at nine days postinfection of *Ae*. *albopictus* mosquitoes with chikungunya virus, yet that population is not seen at three days postinfection [33]. In contrast, mosquitoes infected with dengue virus type 2 show the highest accumulation of vsiRNAs at nine days postinfection, whereas piRNA-sized reads are the dominant population at two days postinfection [35]. On the whole, it is currently unclear how differential accumulation of vsiRNAs and vpiRNAs during the course of infection shapes the immune response in mosquitoes. An intriguing possibility is that the ratio of these two classes of small RNAs is important for the transition from an acute defense mechanism to the establishment of a persistent infection.

In line with this idea, Goic et al. have proposed a model through which the mosquito piRNA pathway may regulate tolerance against dengue and chikungunya virus in *Aedes* mosquitoes during persistent infections [33]. Central to the proposed mechanism is the production of piRNAs from a viral DNA form (vDNA) of these cytoplasmic RNA viruses (Fig 3). Unlike retroviruses, these viruses do not encode their own reverse transcriptase necessary for the generation of a DNA form. Instead, it is thought that cDNA production depends on the reverse transcription activity of endogenous retrotransposons, a mechanism that has been demonstrated previously in *Drosophila* [79]. Administration of a reverse transcriptase inhibitor causes reduction of both vsiRNA and vpiRNA levels, suggesting that a viral cDNA form is required for the establishment of effective small RNA responses. Mosquitoes treated with reverse transcriptase inhibitors die faster after virus inoculation without a strong increase in viral loads. Therefore, the authors conclude that the production of viral cDNA is important for tolerance to virus infection [33]. Yet, the molecular mechanisms linking vpiRNA production and this tolerance phenotype require further investigation. It is possible that vDNA, either integrated in the host genome or existing as episomal sequences, give rise to aberrant transcripts that are processed into piRNAs. Additionally, genomic integration of vDNA close to transposable elements may favor recognition of vDNA-derived transcripts by the piRNA machinery.

Many viruses have developed strategies to evade or interfere with antiviral pathways. For instance, several insect viruses have evolved mechanisms to suppress various steps of the antiviral siRNA pathway [8,80]. Likewise, if the piRNA pathway exerts strong antiviral activity, it is likely that arboviruses have evolved suppressors of piRNA biogenesis and function. Intriguingly, introduction into the chikungunya genome of the gene encoding the Flock House virus B2 protein, an established suppressor of the siRNA pathway, results in a slight decrease of vpiRNA levels [30]. Whether this is due to direct interference with the piRNA pathway or to indirect effects (for example, by affecting RNA abundance or accessibility) remains unclear.

## piRNAs and Endogenous Viral Elements: Heritable Immune Memory?

The canonical function of piRNAs is to provide heritable immunity against transposable elements. The piRNA machinery is able to adapt to newly introduced transposable elements when these integrate into genomic piRNA clusters [81]. In germ cells, these integration events are heritable and therefore provide an evolutionary benefit. It is an intriguing hypothesis that the piRNA pathway in mosquitoes, besides providing memory of transposon encounters, may establish heritable immunity against circulating viruses.

Strikingly, remnants of cytoplasmic RNA virus genomes are frequently integrated in genomes of host species, thus providing a record of previous virus encounters [82,83]. These endogenous viral elements (EVE) may contribute to antiviral immunity in both invertebrates and vertebrates. For example, the genome of the ground squirrel accommodates a large number of endogenous bornavirus-like N elements (EBLN), which are commonly integrated in mammalian genomes [84]. Some EBLNs contain intact open reading frames, and expression of the encoded proteins interferes with infection with a related virus [85]. Besides the expression of viral proteins from EBLNs, piRNAs have recently been hypothesized to contribute to the EBLN-mediated immunity in the mammalian germline [86].

Strikingly, *Aedes* genomes contain a large number of EVEs, some of which are annotated as protein-coding ORFs in the published genome assembly [49,87–90]. PCR-based surveys show that mosquito populations differ in EVE content, indicating that EVEs may be dynamically acquired and stably inherited to the next generation [87–89]. Intriguingly, mosquito EVEs are a prominent source of piRNAs [49]. These piRNAs are mostly antisense to the orientation of the putative viral ORFs [49], suggesting an evolutionary benefit in retaining EVEs that produce piRNAs with the potential to target cognate viral protein-coding RNA. Yet, the extent to which these EVE-derived piRNAs represent a heritable antiviral immune memory needs to be explored.

Interestingly, RNA-mediated antiviral resistance had previously been demonstrated in adult mosquitoes and cells. Expression of genome segments of dengue or La Crosse virus prior to infection with the same viruses interfered with virus replication [76,91–93]. Mutagenesis of in-frame start codons in the expressed viral sequence did not alter this resistance phenotype, indicating that it was mediated by RNA [92]. Moreover, the expression of viral sequences provided partial cross-protection, since replication of related viruses but not viruses from a distinct family was inhibited [91,92]. Similarly, in an attempt to gain siRNA-mediated immunity against dengue virus, Adelman et al. generated clonal C6/36 cell lines harboring a plasmid-encoded inverted repeat to produce dsRNA targeting the dengue prM gene. A highly resistant cell line was obtained, and the authors attributed this resistance phenotype to the production of viral siRNAs. Indeed, production of small RNAs with dengue sequences was shown by northern blotting [94]. However, later studies found that C6/36 cells are Dicer-2 deficient and therefore incapable of producing siRNAs [28]. It is tempting to speculate that the observed dengue resistance was in fact mediated by piRNAs.

Another small RNA-mediated pathway that provides immune memory through integration of foreign genetic information into the genome is the CRISPR-Cas system. In the prokaryotic CRISPR system, short spacer sequences derived from foreign genetic material are incorporated in designated genomic loci. These spacer sequences guide CRISPR-associated (Cas) proteins to exogenous target sequences and as such provide heritable immunity against viruses and plasmids [95]. The piRNA pathway has many similarities with the CRISPR system; in both systems, exogenous nucleic acid sequences are found in specific clusters, which produce small RNAs that guide proteins with endonucleic activity to cognate target sequences [26,49,96]. Despite their obvious similarities, there are also major differences between the two RNA-guided silencing pathways. While in the CRISPR system newly acquired spacers are incorporated in an orderly fashion, incorporation of novel sequences into piRNA clusters depends on retrotransposon activity and appears to be random. Hence, adaptation to new threats is thought to be less efficient in piRNA clusters than in CRISPR loci [81]. Nonetheless, the possibility that piRNA clusters may encode a heritable immune memory in vector mosquitoes similar to the prokaryotic CRISPR system is intriguing and solicits further investigation.

## vpiRNAs in Other Host Species

Whereas vpiRNAs can be readily detected in *Aedes* mosquitoes and cell lines, vpiRNAs have thus far not been reported in important blood-feeding mosquito vectors from the *Anopheles* and *Culex* genera. The *Anopheles gambiae* genome encodes, like *Drosophila*, two orthologs of Piwi/Aub and one copy of Ago3. The *Cx*. *quinquefasciatus* PIWI gene family, however, has undergone expansion to seven members [40,41].

Infection of *An*. *gambiae* with o’nyong-nyong virus (*Alphavirus* genus, *Togaviridae* family) does not give rise to an abundant population of piRNA-sized small RNAs [97]. Yet, in this study, the authors did not analyze additional piRNA features of the small amount of piRNA-sized reads in the sequencing libraries, making it hard to conclusively exclude low level vpiRNA production. Since related viruses give rise to ping-pong amplified vpiRNAs in *Aedes* mosquitoes, it would be interesting to investigate whether a ping-pong signature is also present for o’nyong-nyong piRNA-sized reads. This may also provide an explanation for the observed increase of o’nyong-nyong virus upon depletion of Ago3 in *An*. *gambiae* mosquitoes [15].

Small RNA deep-sequencing in *Cx*. *pipiens* mosquitoes infected with West Nile virus (WNV) or Usutu virus (*Flavivirus* genus, *Flaviviridae* family) did not uncover vpiRNAs, whereas vsiRNAs were readily detected [98,99]. Whether this is due to *Cx*. *pipiens* being unable to produce vpiRNAs or the inability of WNV to trigger vpiRNA production is unclear, especially as WNV also failed to induce vpiRNA production in *Ae*. *albopictus* C6/36 cells [28], which are competent in producing vpiRNAs from other flaviviruses. In contrast, Sindbis virus infection of *Aedes* cells gives rise to an abundant population of vpiRNAs [29,32] yet fails to induce vpiRNA production in *Culex* mosquitoes (S1 Fig). Thus, although PIWI gene duplications have occurred both in *Aedes* and *Culex*, only *Aedes* PIWI proteins seem to support efficient vpiRNA biogenesis. A possible explanation for this discrepancy would be that *Culex* PIWI genes are not coexpressed with viral RNA in somatic cells. Alternatively, viral RNA might not be a favorable substrate for *Culex* PIWI proteins. Future research will have to characterize to what extent vpiRNA production is supported in different blood-feeding mosquito species.

The piRNA pathway is not frequently studied in insects other than mosquitoes and fruit flies. Nevertheless, PIWI gene duplication and somatic expression of PIWI proteins has been observed in the pea aphid *Acyrthosiphon pisum* [100]. This indicates that there is potential for functional innovation and perhaps viral piRNA biogenesis beyond mosquitoes. Likewise, although PIWI proteins are generally highly expressed in germline tissues in vertebrates, emerging evidence suggests that PIWI proteins may also be expressed in somatic cells including neurons, cancer cells, and stem cells [101,102]. However, it is not yet known whether these somatically expressed PIWI proteins are capable of targeting viral RNA.

## Open Questions

Despite the progress in our understanding of vpiRNA biogenesis and function, many important questions remain: (i) Which mosquito species are capable producing vpiRNAs and which viruses elicit a piRNA response? In relation to these questions, future research should investigate to what extent the piRNA pathway determines vector competence and the specificity of arboviruses for certain mosquito species. (ii) What is the composition of macromolecular complexes required for piRNA production from various RNA sources? It is of particular importance to investigate which PIWI proteins are required for piRNA production from different arboviruses as well as from transposons and other endogenous sources. Also, the contribution of additional proteins to piRNA biogenesis and function warrants investigation. (iii) What is the role of the mosquito piRNA pathway in mediating resistance to and tolerance for arbovirus infections? (iv) What is the contribution of endogenous viral elements to antiviral immunity and immune memory in mosquitoes? (v) Have arboviruses developed strategies to evade or interfere with the piRNA pathway? (vi) How widely do somatic piRNA pathways occur across the tree of life, and has piRNA-mediated gene silencing acquired additional functions beyond transposon control in other animal species? The mosquito piRNA pathway and in particular the production of vpiRNAs shows that the piRNA pathway is much more versatile than previously anticipated. It remains to be seen how many more surprises PIWI proteins have in store when we take a closer look at this fascinating pathway in other species.

## Data Availability Statement

New small RNA sequencing data have been deposited in NCBI Sequence Read Archive (accession number SRA486748).

## Supporting Information

S1 Fig Size profile of Sindbis virus small RNAs in *Culex pipiens*. *Cx*. *pipiens* mosquitoes were infected with 9,660 TCID_50_ Sindbis virus (pTE 2J 3′GFP) by intrathoracic injection. Two days postinfection, RNA was extracted from the mosquitoes using Isol-RNA lysis reagent. Small RNAs were isolated by gel-electrophoresis, and deep-sequencing libraries were prepared using Illumina’s Truseq small RNA preparation kit. Small RNA libraries were then sequenced on a Illumina Hiseq2500 system and mapped to the Sindbis virus genome. The size distribution of viral small RNAs derived from the sense strand (black) or antisense strand (grey) is depicted for sequencing reads that align to the genome with a maximum of one mismatch in the first 28 nt. The size profile suggests that *Cx*. *pipiens* does not produce vpiRNAs, but it cannot be excluded that vpiRNAs are found when using a different route of inoculation, at other time points, or in infections with other viruses. Deep-sequencing data have been deposited in the NCBI Sequence Read Archive under accession number SRA486748.
(EPS)
